# The Effect of Ryegrass Silage Feeding on Equine Fecal Microbiota and Blood Metabolite Profile

**DOI:** 10.3389/fmicb.2021.715709

**Published:** 2021-08-23

**Authors:** Yiping Zhu, Xuefan Wang, Bo Liu, Ziwen Yi, Yufei Zhao, Liang Deng, Reed Holyoak, Jing Li

**Affiliations:** ^1^Equine Clinical Diagnostic Center, College of Veterinary Medicine, China Agricultural University, Beijing, China; ^2^College of Animal Husbandry and Veterinary Medicine, Shenyang Agricultural University, Shenyang, China; ^3^College of Veterinary Medicine, Oklahoma State University, Stillwater, OK, United States

**Keywords:** fecal microbiota, hay, horses, metabolome, silage

## Abstract

Silage is fed to horses in China and other areas in the world, however, knowledge about the impact of feeding silage on horse health is still limited. In the current study, 12 horses were assigned into two groups and fed ryegrass silage and ryegrass hay, respectively, for 8 weeks. High-throughput sequencing was applied to analyze fecal microbiota, while liquid chromatography–tandem mass spectrometry (LC–MS/MS) based metabolomics technique was used for blood metabolite profile to investigate the influence of feeding ryegrass silage (group S) compared to feeding ryegrass hay (group H) on equine intestinal and systemic health. Horses in group S had significantly different fecal microbiota and blood metabolomes from horses in group H. The results showed that Verrucomicrobia was significantly less abundant which plays important role in maintaining the mucus layer of the hindgut. *Rikenellaceae* and *Christensenellaceae* were markedly more abundant in group S and *Rikenellaceae* may be associated with some gut diseases and obesity. The metabolomics analysis demonstrated that ryegrass silage feeding significantly affected lipid metabolism and insulin resistance in horses, which might be associated with metabolic dysfunction. Furthermore, Pearson’s correlation analysis revealed some correlations between bacterial taxa and blood metabolites, which added more evidence to diet-fecal microbiota-health relationship. Overall, ryegrass silage feeding impacted systemic metabolic pathways in horses, especially lipid metabolism. This study provides evidence of effects of feeding ryegrass silage on horses, which may affect fat metabolism and potentially increase risk of insulin resistance. Further investigation will be promoted to provide insight into the relationship of a silage-based diet and equine health.

## Introduction

As a non-ruminant grazing herbivore, horses have undergone domestication and been adapted to eating forage-based diets with a mixed symbiotic microbiota (De Fombelle et al., 2003). Gut microbial flora plays critical roles in animal health by extracting energy from the diet for growth, guarding the gastrointestinal system as a commensal barrier and modulating gut mucosal immunity (Shulman et al., 2008; Cox et al., 2014). Dietary composition undoubtedly impacts the intestinal ecosystem. Therefore, inappropriate diet management can also be a risk factor for intestinal diseases such as colic (Tinker et al., 1997). There is ever growing knowledge of the impact of different diets and feeding patterns on the microbial signatures of the horse gastrointestinal system (De Fombelle et al., 2001; Daly et al., 2012; Venable et al., 2017). Characterization of gut microbiota in horses fed forage, hay, high-starch diet, as well as different supplements such as cereal and oil have been reported (Dougal et al., 2014; Fernandes et al., 2014; Sorensen et al., 2021). Profound impact of the different diets on horse gut microbiota has been revealed.

Traditionally, horses were able to graze fresh pastures. However, nowadays many of them are fed preserved forages, including hay, haylage and silage, due to environmental limits on grazing and modern management systems (Glunk et al., 2015; Harris et al., 2017). Silage is a type of fermented forage preserved moist and airtight with more volatile products compared to hay (Han et al., 2006). In European countries, the use of silage as feed for horses is increasing since this type of diet is easier to store (Vandenput et al., 1997; Muhonen et al., 2008). Silage is also fed to horses in some areas of China, such as Xinjiang province, for economic reasons, convenience of harvesting, and some nutrition components, including volatile fatty acids (VFA) (Guo, 2014; Harris et al., 2017). Ryegrass was widely planted in Asia, Europe and America due to its high nutritional value (Parvin et al., 2010). It has been popular forage crop for large animals in China (Li et al., 2019), therefore, ryegrass was used as silage and hay source in this study. A few studies have reported effects of silage on the equine intestinal ecosystem, and it has been considered a cause of gut microbial flora disturbances, such as soft stools in horses (Holmquist and Müller, 2002). In Muhonen’s study, the disturbance was explained as an abrupt change of diet, instead of silage feeding and only a small decrease in pH and an increase in VFA were observed (Muhonen et al., 2008). In a recent study, no significant impact was found on intestinal health of horses fed silage for 8 weeks, even though significant microbial differences were revealed between horses fed silage, hay and pasture grasses (Zhu et al., 2021). Nonetheless, the marked impact of diet induced gut microbial changes on horse general health remains unclear.

Gut microbiota is not only influenced by many host factors but also can modulate metabolic phenotypes (Armstrong et al., 2017). Microbial candidates strongly associated with host metabolic pathways are likely to be more relevant to host health, for example, *Faecalibacterium prausnitzii* population is associated with host energy metabolism and mucosal integrity indicating its critical role in host gastrointestinal health (Li et al., 2008). There is an increased effort to study diet-metabolite-health relationships in humans and other species including horses (Sun et al., 2016; Guasch-Ferré et al., 2018; Sanguinetti et al., 2018). Human based studies focusing on the multilevel dimensional complexity of diet-microbiome-host interactions have provided more insights into the impact of gut microbiota on systemic health via the production of metabolites (Marchesi et al., 2016). There is evidence that host gut microbiota is profoundly involved in many metabolic pathways, such as lipid metabolism and amino acid synthesis (Li et al., 2008). Nevertheless, the understanding on the interactions among different dietary patterns, gut microbiota and health via metabolite production is still relatively new even in humans (Tindall et al., 2018).

In this study using high-throughput omics approaches, we investigated the interaction between the horse fecal microbiota and blood metabolite profile, when fed two diets (hay and ryegrass silage). Silage feeding to horses has been a hot topic in Chinese equine industry for years and this is the first study about the effect of silage diet on systemic metabolism and health in horses besides fecal microbiota. It is intended to facilitate understanding on health outcomes of horses fed different diets especially silage.

## Materials and Methods

### Animal and Ethics Statement

All procedures involving animals in this study were carried out with welfare license (No. AW11101202-2-1) issued by Animal Care and Use Committee of the China Agriculture University.

### Experimental Design and Sample Collection

Twelve light breed (Guanzhong) horses located in the in Shanxi Province, including eight mares and four stallions with normal body condition scores (BCS 4–6, mean = 4.8), were selected for this study. Body weight of each horse was estimated using body weight formula developed by Carroll and Huntington (1988) and the average weight was 389 kg (SD ± 19 kg). A complete physical exam was performed on each animal before the initiation of the experiment. All were clinically healthy. Horses involved in this study had no history of illness or medical treatments in the 6 months prior to its initiation. The horses varied in age from 3 to 13 years, with a mean age of 6.9 (SD ± 2.7). Prior to this study, all horses were kept in individual stalls and fed with mainly commercial hay and a small amount of home-made corn-based concentrates formulated locally.

The diets involved in this study were either ryegrass hay (local pasture ryegrass, 15% dry matter [DM]) or ryegrass silage (26% DM). Hay used in this study was second cutting ryegrass from local pasture (September–October) and ryegrass silage was soft-dough-high stage cut from the pasture in the area. The nutritional analysis has been performed on both types of forages (Table 1), which was previously described (Zhu et al., 2021). The eight mares and the four stallions were randomly and equally divided into two groups, respectively. One group of mares and one group of stallions were combined in a random way as well with six horses (four mares and two stallions) in each group. One was fed ryegrass silage (group S) and one fed ryegrass hay (group H). Each group fed ryegrass silage or hay, respectively started at 0800 h on the same day, lasting for 8 weeks as previously described (Zhu et al., 2021). To minimize gut disturbances there was a gradual introduction and acclimatization of the new diets which was completed in 1 week. Each horse was fed a daily maintenance ration of 2% of body mass on a DM basis (Dugdale et al., 2010). Body weight estimation and physical exam were performed weekly on each horse. All horses had access to water *ad libitum*, with no other dietary supplements throughout the experiment.

**TABLE 1:** Nutritional composition: ryegrass silage, hay (As fed).

	**Ryegrass silage**	**Ryegrass hay**
DM^1^ (%NM^2^)	25.78	91.50
CP^3^ (%DM)	8.54	9.36
CF^4^ (%DM)	1.34	1.82
Ash (%DM)	9.91	5.04
NDF^5^ (%DM)	66.62	59.89
ADF^6^ (%DM)	46.94	34.71
Ca^7^ (%DM)	0.28	0.16
P^8^ (%DM)	0.22	0.06

*1 dry matter; 2 natural matter; 3 crude protein; 4 crude fat; 5 neutral detergent fiber; 6 acid detergent fiber; 7 calcium; 8 phosphorus.*

Fecal samples were collected manually from the rectum of all horses prior to the onset of the feed change and again at the end of the 8-week feeding phase as described previously (Zhu et al., 2021). Each fresh fecal sample was saved in a clean self-sealing bag and immediately placed on ice for transportation (approximately 2 h) to the lab. Upon arrival at the laboratory, the center of each fecal ball was collected sterilely (Stewart et al., 2018) and transferred to a 2 mL sterile cryogenic vial (Corning, Corning, NY, United States). Each vial was marked and stored at –80°C right after center collection for further analysis.

Meanwhile, one set of blood samples were collected from each horse at the end of the trial. Eight milliliter blood was drawn from the horse’s jugular vein into a pro-coagulation tube (Sanli Medical and Technological Development Co., Ltd., Liuyang, China) and immediately placed upright in ice. The serum of each blood sample was centrifuged and stored at −80°C right away until further analysis.

### Fecal Sample Processing and 16S rRNA High-Throughput Sequencing

Bacterial DNA from each fecal sample was extracted using a E.Z.N.A.^®^ soil DNA Kit (Omega Bio-tek, Norcross, GA, United States) in accordance with manufacturer’s instructions. DNA concentration and purity were assessed after extraction. High-throughput 16S rRNA sequencing was then performed to evaluate the composition and diversity of the bacterial community.

The V4 hypervariable region of the 16S rRNA genes was amplified using 515F (5′-GTGCCAGCMGCCGCGGTAA-3′) and 806R (5′ GGACTACHVGGGTWTCTAAT-3′) primer pairs. Positive template and negative control of distilled water were also employed. After PCR amplification, products were electrophoresed and purified using an AxyPrep DNA Gel Extraction Kit (Axygen Biosciences, Union City, CA, United States) following manufacturer’s recommendations. PCR products were quantified using a Quantus Fluorometer (Promega, Madison, WI, United States). Purified amplicons pooling and paired-end sequencing were performed with Illumina MiSeq PE300 platform and NovaSeq PE250 platform (Illumina, San Diego, CA, United States). Raw 16S gene sequence data was quality-filtered using FASTQ (v 0.20.0.) (Chen et al., 2018). The sequencing reads merge was performed with FLASH (v 1.2.7) (Magoč and Salzberg, 2011). Operational taxonomic units (OTUs) were clustered at a 97% similarity level (Stackebrandt and Goebel, 1994) using UPARSE (v 7.1) (Edgar, 2013). Each OTU taxon was evaluated for the most abundant sequences by QIIME (v 1.9.1) (Caporaso et al., 2010; Brandwein et al., 2019) and analyzed for the representative sequence against the Silva rRNA database (v 138) (Quast et al., 2013) with the Ribosomal Database Projection (RDP) Classifier (v 2.2.0) (Wang et al., 2007).

### Serum Sample Processing and Untargeted Metabolic Analysis

Untargeted metabolic analysis was performed on serum samples from each horse using liquid chromatography–tandem mass spectrometry (LC–MS/MS) system (Merware, Wuhan, China). To extract hydrophilic compounds, 50 μL Serum samples were added to 300 μL pure methanol after having been thawed on ice. The mixture was vortexed for 3 min, then centrifuged with 12,000 rpm at 4°C for 3 min. A 150 μL sample of supernatant was taken for LC–MS/MS analysis. Hydrophobic compounds were extracted by homogenizing with 1 mL mixture (including methanol, MTBE and internal standard mixture), centrifuging with 12,000 rpm at 4°C for 10 min and supernatant dissolving with 200 μL mobile phase B. Sample extracts were then analyzed using ultra performance liquid chromatography (UPLC) and tested for ion mode by electrospray ionization (ESI).

### Statistical Analysis

Alpha diversity analysis, including Chao index representing richness (number of taxonomic groups; Willis, 2019) and Shannon index illustrating diversity (the variety and abundance of taxonomic groups; Magurran, 2004), were conducted using MOTHUR (v 1.3.0). Subsampling was performed to normalize the dataset and pair-wise comparisons between groups were performed by the Student’s *t*-test. Principal component analysis (PCA) and Principal coordinates analysis (PCoA) were carried out using QIIME (v 1.9.1). Linear discriminant analysis (LDA) effect size (LEfSe) (Segata et al., 2011) was performed by the Kruskal-Wallis sum-rank test to determine significant differences in abundance of the microbial community between the groups.

Significantly regulated metabolites between groups were detected by variable-importance-projection (VIP) values ≥ 1.0, hypergeometric test’s *p*-values < 0.05 and absolute Log_2_FC (fold change) ≥ 1.0. VIP values were extracted from on orthogonal partial least squares discriminate analysis (OPLS-DA) result using MetaboAnalystR (v 1.0.1) (Edmonton, CA, United States). Metabolites set enrichment analysis (MSEA) was performed for pathways with significant regulated metabolites. Heatmaps were generated using Pearson’s correlation coefficients (PCC) with ComplexHeatmap (v 3.5.0) (Gu et al., 2016). Identified metabolites were annotated by Kyoto Encyclopedia of Genes and Genomes (KEGG) compound database and mapped to the KEGG pathway database.

## Results

Throughout the 8-week feeding phase the average body condition score and body weight of each horse did not change significantly. Weekly physical exam was unremarkable of each horse and no clinical abnormalities have been noted throughout the feeding trail.

### Summary of 16S rRNA Amplicon Sequencing Data

A total of 810,491 sequence reads were detected from all the samples. With 731,179 sequences, ranging from 44,873 to 71,449 per sample, remaining after quality control. After clustering at the 97% threshold level, 3,191 OTUs were retained and classified as bacteria within 25 phyla, 50 classes, 118 orders, 209 families, and 423 genera. The raw data were deposited into the NCBI Sequence Read Archive (SRA) database (Accession Number: PRJNA735708).

### Effects of Diet on Microbial Composition

**FIGURE 1:**
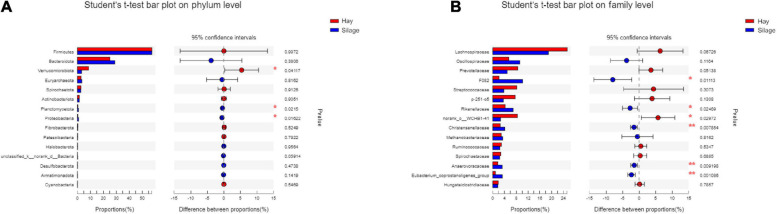
Relative abundance of the dominant bacterial taxa (> 1%) at the phylum **(A)** and family **(B)** level of two groups. Student’s *t*-test was used to assess the difference of the relative abundance between the two groups. *P < 0.05, **P < 0.01. Red, group H; Blue, group S.

Six phyla and twenty-three families of bacterial taxa had relative abundance > 1%. The six most abundant phyla were Firmicutes, Bacteriodetes, Verrucomicrobia, Spirochaetes, Euryarchaeota, and Actinobacteria (Figure 1A). Only the relative abundance of Verrucomicrobia was significantly different between the two groups (*P* = 0.03943). The level of Verrucomicrobia was higher in group H than in group S. At the family level among the top 10 most abundant bacterial taxa, *Rikenellaceae* and *Christensenellaceae* were notably affected by diet (*P* < 0.05), with both detected at a higher level in group S (Figure 1B).

**FIGURE 2:**
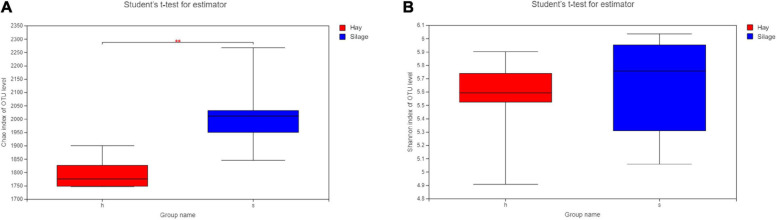
Alpha diversity indices of the fecal microbiota in horses fed silage (S, *n* = 6) and hay (H, *n* = 6), **P < 0.01. **(A)** Bacterial taxa richness or Chao index of each group **(B)** Bacterial taxa diversity of Shannon index of each group. Red, group H; Blue, group S.

**FIGURE 3:**
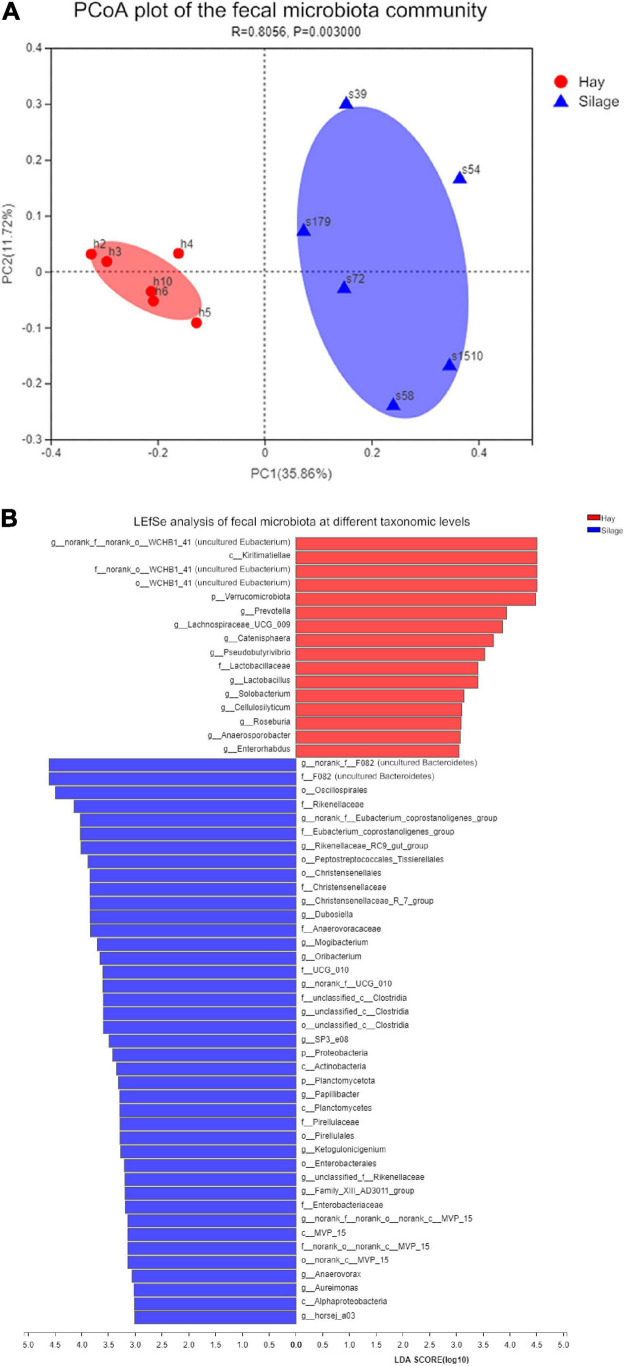
Microbial community analysis of fecal microbiota in group S and group H. **(A)** Principal coordinates analysis (PCoA) was conducted based on Bray-Curtis distances with *P*-values of Anosim to emphasize the difference of fecal microbiota community in the two groups. Red dot: PCoA value of individual horse in group H, blue triangle: PCoA value of individual horse in group S. **(B)** LEfSe illustrates the difference of fecal microbiota between group S and group H at different levels. only taxa with LDA score of 3 or above are shown and ranked by the effect size in LEfSe. Red, group H; blue, group S.

Alpha diversity analysis including richness (Chao index) and diversity (Shannon index) of microbial species was conducted based on the Student’s *t*-test. The Chao index showed the microbial composition of group S was significantly richer than that of group H (*P* < 0.01) (Figure 2A), while the Shannon index revealed no statistical difference in microbial diversity between the two groups (Figure 2B). The PCoA, based on the Bray-Curtis distance, demonstrated two separate clusters, representing group S and group H, respectively (Figure 3A). Additionally, to evaluate the variations of the microbial composition between two groups, LEfSe was performed with LDA score > 2.0. Sixteen taxa were most associated with group H, while forty-two were associated with group S (Figure 3B). In the samples from group S, excluding the undefined taxa, the most distinct bacterial taxa included Oscillospirales, *Rikenellaceae*, and *Eubacterium coprostanoligenes*. In group H, the two most associated bacterial taxa were Kiritimatiellae and Verrucomicrobia.

### Effects of Diet on Metabolite Profiles

**FIGURE 4:**
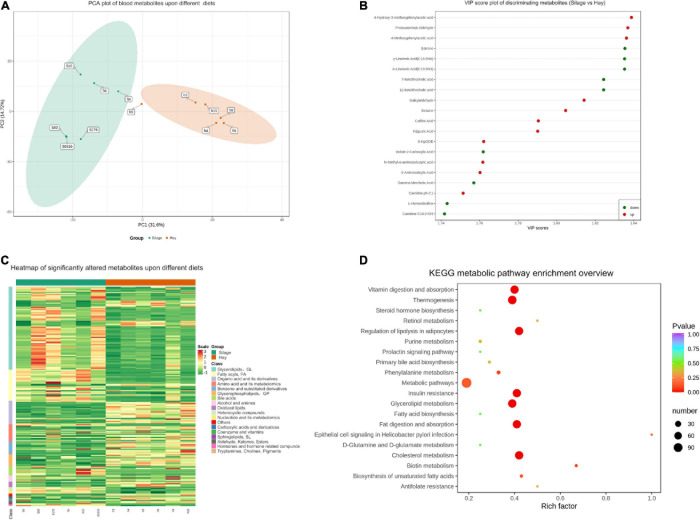
Effect of ryegrass silage on horse metabolite profiles. **(A)** Principal component analysis (PCA) of blood metabolites of group S and group H. Green dot: PCA value of individual horse in group S. Orange rectangle: PCA value of individual horse in group H. **(B)** Metabolites discriminating between group S and group H with VIP > 1 were listed. Red dots indicate up-regulated metabolites. Green dots indicated down-regulated metabolites. **(C)** Heatmap of significantly altered metabolites in group S and group H. Individual samples (horizontal axis) and metabolites (vertical axis) are presented using hierarchical cluster analysis. The color scale is noted on the right side of the figure. Red and green color represent up-regulated and down-regulated metabolites, respectively, relative to the median metabolite level. **(D)** KEGG enrichment of each pathway related to this study was presented using rich factor. *P*-values and impact values are indicated on the horizontal axis and vertical axis, respectively. The sized and colors of the shapes represent the influence of silage diet on sample pathways relative to hay diet. The larger red shapes indicate greater impact of the diet on the pathway.

To evaluate the effects of ryegrass silage and hay diets on the horse’s metabolite production, untargeted metabolic analysis by LC-MS/MS was performed. Before differential analysis of the groups of metabolites, PCA analysis was conducted to illustrate the sample variation between groups and within groups (Figure 4A). Samples from group S and group H were well separated. Furthermore, the horses fed ryegrass silage demonstrated larger metabolite composition variation as compared to horses on the hay diet.

Significant changes in metabolites between group S and H were detected according to the following criteria: VIP values > 1.0 based on OPLS-DA, fold change > 2 or < 0.5 or *P* < 0.05. In total, 206 discriminating metabolites were selected from the two groups. In group H, eighty-two compounds were up-regulated including estrone, γ- and α-Linolenic acid, 7- and 12-ketolithocholic acid (Figure 4B). Whereas 124 metabolites were down-regulated in group H as compared to group S, including 4-Hydroxy-3-methoxyphenylacetic acid, protocatechuic aldehyde, 4-methoxyphenylaceti acid, and most types of triglycerides belonging to glycerolipids (Supplementary Table 1). A heat map was produced to show the great variation in their distribution within each group (Figure 4C). Eighteen metabolites were shown to have significantly different distributions between group S and group H. Glycerolipids (mainly triglycerides) and fatty acids (mainly γ-linolenic acid, palmitoleic acid, stearidonic acid, and carnitine) were the most abundant metabolites in group S, while being notably lower in abundance in group H. Organic acids and derivatives, amino acids and metabolomics, benzene and substituted derivatives, as well as glycerophospholipids, were more abundant in group H than in group S.

To explore the biological functions of the metabolites in the two groups, KEGG enrichment analysis was conducted (Figure 4D). Seven significantly different enriched pathways (*P* < 0.05) were recognized including cholesterol metabolism, thermogenesis, regulation of lipolysis in adipocytes, insulin resistance, fat digestion and absorption, vitamin digestion and absorption and glycerolipid metabolism.

### Correlation Between Microbial Community and Blood Metabolites

**FIGURE 5:**
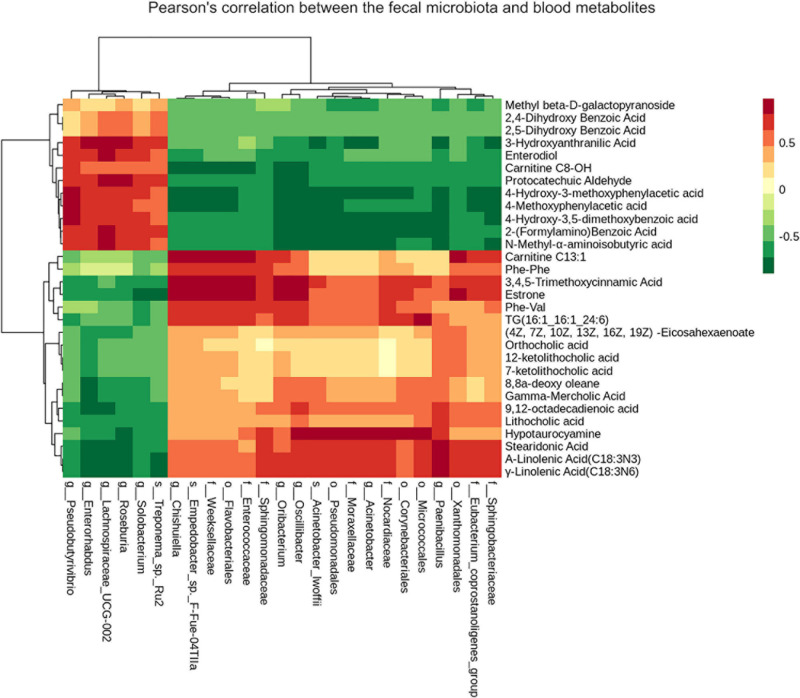
Heatmap of Pearson’s correlation between the fecal microbiota and blood metabolites significantly different between groups is illustrated. Red represents significantly positive correlation (*P* < 0.05), green represents significantly negative correlation (*P* < 0.05) and yellow represents that the correlation is not significant (*P* > 0.05).

To further explore the correlation between blood metabolites and the altered fecal microbiota as induced by the two diets, analyses based on the Pearson’s correlation coefficients were conducted (Figure 5). Discriminating metabolites were selected based on a fold change of > 2 or < 0.5, while altered microbial taxa were identified using the *P*-value < 0.05. These analyses revealed high correlations between some bacterial taxa and typical metabolites. For instance, *Eubacterium coprostanoligenes* were positively correlated with triglycerides and fatty acids such as stearidonic acid, α-linolenic acid and γ-linolenic acid, but negatively correlated with organic acids and phenolic acids, such as 4-methoxyphenylacetic acid, 3-hydroxyanthranilic acid and 2,4-dihydroxy benzoic acid. Triglycerides were positively correlated with *Sphingobacteriaceae*, *Eubacterium coprostanoligenes*, and *Paenibacillaceae*, however, negatively correlated with *Treponema, Roseburia* and *Lachnospiraceae*.

## Discussion

Silage is often used as horse feed in China and other countries, especially in Europe (Muhonen et al., 2008; Domingues, 2009; Guo, 2014), however, occasional disturbances, such as soft or loose stool, have been reported when silage has been introduced as a feed stuff (Connysson et al., 2006). The available information about the influence of silage on equine gut and systemic health is limited. Therefore, the objectives of this study were to explore how the two different diets, including ryegrass silage and ryegrass hay, would modulate fecal microbiota and blood metabolomics, indicating the potential association of silage with intestinal and systemic level of health. Characterization of the blood metabolome, associated with fecal microbiota, highlights the effects of diet on systemic biological molecules and provides useful insights into the critical role of the gastrointestinal tract in maintaining general equine health. The results of fecal microbiota and blood metabolites have shown significant differences between horses fed ryegrass silage compared to being fed ryegrass hay. It will be more informative of the silage impact on horse gut and systemic health if larger number of horses and more types of silage or forages are involved in the future.

In horses, gut microbiota has been widely studied by high-throughput sequencing technique, therefore, the complexity of microbial communities can be demonstrated with the high depth of coverage of OTUs (Di Bella et al., 2013). However, since obtaining samples of intestinal ingesta is very difficult, fecal matter is used as a replacement for gut material (Julliand and Grimm, 2017). Fecal microbiota is sensitive to dietary variations and can be representative of the major hindgut sections (Julliand and Grimm, 2016). Therefore, this study used fecal samples to investigate the influence of silage and hay on hindgut microbiota in horses. Silage and hay used in this study were both made out of ryegrass from local pasture which could be a representative of the most commonly used forage in China. Analysis on more types of silages will be helpful to better illustrate the impact of feeding silage on horse health. In the current study, we found that microbial richness in fecal samples from group S were significantly higher than that in group H. However, no difference in the diversity of fecal microbiota was identified. It has been reported that an increase in the concentration of total anaerobic bacteria was observed on day 14 and 22 after the onset of feeding silage to horses. However, this was attributed to variations of sampling time, instead of an impact from the diet (Muhonen et al., 2008). In this study, increased fecal microbial richness induced by silage-feeding is consistent with the previous research (Muhonen et al., 2008). Further investigations on the chemical components in silage which may have contributed to our findings will facilitate a greater understanding of the association between silage diet and gut health in horses.

The relative abundance of Verrucomicrobia was higher in group H than in group S. Besides, it was also identified as the most discriminating phylum of group H based on LEfSe results. In previous studies, it is one of the most common and abundant phyla detected in the equine hindgut microbial ecosystem (Costa et al., 2012; Shepherd et al., 2012; Steelman et al., 2012). Verrucomicrobia is a phylum consisting mainly of strict anaerobes, considered to play an important role in hindgut function, for example, maintaining the mucus layer between the gut lumen and enterocytes (Arnold et al., 2020). Remarkably, a decreased abundance of Verrucomicrobia has been reported in horses with gastrointestinal disturbances after metronidazole administration (Arnold et al., 2020) and in horses with laminitis (Steelman et al., 2012). Therefore, Verrucomicrobia is a good candidate to study for its impact on metabolic function. Further research will be necessary to investigate whether a lower abundance of Verrucomicrobia in ryegrass silage-fed horses is associated with a disruption of the gut ecosystem and associated systemic sequelae. Even though Kiritimatiellae was dominantly associated with horses on hay diet, there is not much information reported on its role or function in equine gut microbiota.

Statistically higher abundance levels of *Rikenellaceae* and *Christensenellaceae* were demonstrated in group S. *Rikenellaceae* has been reported in several studies of the equine microbiota (Biddle et al., 2018; Mach et al., 2020). *Rikenellaceae* is a mucin-degrading bacteria (Sivaprakasam et al., 2019) and mucin degradation has been shown to be a normal turn-over process of the colon epithelium (Norin et al., 1985). In an investigation of gut flora modulation by the probiotic *Lactobacillus* in mice, *Rikenellaceae* was shown to decline along with an increase of *Lactobacillus* (Xing et al., 2018) and high level of *Rikenellaceae* further associated with pathologic diseases, such as inflammatory bowel disease (Alkadhi et al., 2014) and obesity in other species (Kim et al., 2012). In accordance with LEfSe analysis, *Lactobacillus* was more associated with group H whilst *Rikenellaceae* was highly associated with group S. The relative abundances of *Rikenellaceae* in group S and group H were 7.0 and 4.2%, respectively, and *Lactobacillus* levels were 0.2 and 0.8% in group S and group H, which also presented a negative correlation between the two bacterial families in horse fecal microbiota. Even though biological function of *Rikenellaceae* and its relationship with *Lactobacillus* in horse gut are still not well defined, it might still be worth studying in silage-fed horses as one of the characteristic bacterial taxa. *Christensenellaceae* revealed the same trend as *Rikenellaceae*, however, its role in equine gut health requires more investigation.

*Eubacterium coprostanoligenes* is another bacterial family demonstrated significantly different relative abundance between two groups and more associated with group S according to LEfSe analysis. As a member of butyrate-producing bacteria, *Eubacterium coprostanoligenes* has been linked to mitochondrial gene expression involved in energy metabolism pathways including fatty acids (Mach et al., 2021) which was one of the most prominent metabolites in group S. Additionally, *Eubacterium coprostanoligenes* and fatty acids showed positive correlation in correlation analysis between fecal microbiota and blood metabolites. Even though not much investigation has been carried out on its specific role in equine gut microbiota, results in this study indicated that silage feeding could impact level of *Eubacterium coprostanoligenes* and fatty acids. Further study on the influence of silage feeding on gut microbial changes may help understand better about fat metabolism-silage relationship. According to LEfSe, *Oscillospiraceae* was shown associated with horses fed silage, however, further investigation will be necessary to learn more about its relationship with diet and gut health (Zhu et al., 2021).

Plasma and urine samples are considered the most appropriate biofluids for metabonomic analysis in horses due to lower variability in metabolomic results, compared to fecal samples (Escalona et al., 2015). In the current study, serum collected from each horse was used for metabolic analysis. There was a dramatic separation of metabolites between the two groups (Figure 4A), indicating a great influence of diet on systemic responses in horses. Triglycerides (belonging to group glycerolipids) and fatty acids were highly upregulated by ryegrass silage feeding (Figure 4C and Supplementary Table 1). As a critical diagnosis for hyperlipemia in horses, concentration of triglycerides is also used to predict metabolic dysregulation (Jacob et al., 2018). Increased concentrations of circulating fatty acids and triglycerides have been associated with insulin resistance in horses (Carter et al., 2009). In addition, the KEGG enrichment analysis (Figure 4D) identified that insulin resistance was one of the most differentiating pathways between the two groups, which further highlights the potential association of a silage diet and metabolic dysfunction. Fatty acids are also known to activate proinflammatory pathways leading to an increase of proinflammatory cytokines, including tumor necrosis factor alpha and interferon gamma, which interfere with the insulin signaling cascade resulting in a negative impact on insulin sensitivity (Manning, 2004). One study also showed that high levels stearic acid and palmitoleic acid, which were also the two main fatty acids found significantly higher in group S, have been associated with high insulin resistance in humans (Kurotani et al., 2012). Therefore, silage may have negative impacts on metabolic function in the long term. More extensive research studying levels of inflammatory cytokines, inflammation-associated fatty acids, blood insulin as well as lipid level are warranted to provide more evidence on association of silage and equine metabolic disorders. There was another interesting finding in this study that the crude fat (CF) content in group S and group H was 1.34 DM and 1.83% DM, respectively, yet not positively correlated with the lipid-metabolism related metabolites. It could indicate that the impact of silage on lipid metabolism is not associated with the amount of dietary fat but metabolic pathways in the body.

By observing the VIP score plot (Figure 4B) bile acids, such as 7-ketolithocholic acid and 12-ketolithocholic acid, were significantly upregulated in group S horses as well. Bile acids are critical components of lipid metabolism, especially cholesterol, and function mainly in gut (Breuer et al., 1985). Higher bile acids may be a consequence of higher lipid biochemical matrices in horses fed ryegrass silage. It is characteristic in horses that bile acids are secreted continuously, therefore, interpretation of plasma concentrations requires additional relevant information (Toth et al., 2018), for example, a history of being on a silage-based diet, as highlighted by this study. The metabolites most associated with group H horses were relatively more diverse, including organic acids, amino acids and oxidized lipids, which were involved in different metabolic pathways. The biological significance of the many metabolite changes, including 4-Hydroxy-3-methoxyphenylacetic acid, protocatechuic aldehyde and 4-methoxyphenylaceti acid, were beyond the scope of this study. As a result, further controlled studies on the role of silage in horse health may provide deeper understanding of these metabolite functions.

We also investigated the correlation between fecal microbiota and blood metabolomes. Notably, triglycerides and some fatty acids, were positively correlated with the prevalence of the bacterial taxa which were statistically different between the two groups (Figure 5). Most of these bacteria were not very abundant in the horses within this study and only the *Eubacterium_coprostanoligenes* group had a relative abundance of > 1%. It has been positively correlated with triglycerides and fatty acids such as stearidonic acid indicating its potential impact on these metabolites. There were also a few bacterial taxa negatively correlated with triglycerides and fatty acids, such as *Treponema* and *Roseburia*. Therefore, the lower levels of fatty acids and triglycerides were likely attributed to the presence of many bacterial floras at lower relative abundance, instead of just few influential bacterial taxa. The microbiome-metabolome correlation analysis can help recognize relationships between specific types of bacteria and metabolites, which in-turn may indicate a potential mechanism of a disease and their relative impacts on health (Zhao, 2013). However, this type of study alone is not able to determine the origin of the metabolite, as well as the causality between the bacteria and metabolites. As a result, *in vitro* digestion models may be necessary to investigate the relationship between metabolites, diet, and health in horses.

In conclusion, 16S rRNA high-throughput sequencing and untargeted metabolomics helped reveal the fecal microbiota, blood metabolite profile and their correlation with ryegrass silage and hay diets. The major findings of this study are as follows: (a) ryegrass silage and hay lead to significant changes on fecal microbiota. (b) ryegrass silage and hay showed significant impact on blood metabolites composition. (c) ryegrass silage intake revealed association with lipid metabolism and might increase risk of insulin resistance; (d) some specific gut bacterial taxa-related metabolites were strongly correlated with altered gut microbes which indicated great impact of diet on gut and the body system of horses. These results suggest that silage could cause significant changes in the horse fecal microbiota and might be associated with metabolic dysfunctions, such as insulin resistance and lipid metabolic abnormality. Studies measuring level of blood insulin and lipid in horses fed silage will provide more evidence to the association. These findings may promote understanding on the impact of silage feeding on horse health and facilitate making more scientific diet plan for horses especially those with metabolic disorders such as insulin resistance and hypertriglyceridemia.

## Data Availability Statement

The datasets presented in this study can be found in online repositories. The names of the repository/repositories and accession number(s) can be found below: www.ncbi.nlm.nih.gov/, PRJNA735708.

## Ethics Statement

All procedures involving animals in this study were carried out with a welfare license (No. AW11101202-2-1) issued by the Animal Care and Use Committee of the China Agricultural University.

## Author Contributions

JL, YPZ, and RH designed the study. YPZ, XW, ZY, YFZ, LD, and JL participated in animal acquisition and sample collection. XW, LD, and BL helped conduct data analysis. YPZ and ZY contributed to the writing of the original draft. JL, BL, and RH helped review and edit the manuscript. All authors contributed to the article and approved the submitted version.

## Conflict of Interest

The authors declare that the research was conducted in the absence of any commercial or financial relationships that could be construed as a potential conflict of interest.

## Publisher’s Note

All claims expressed in this article are solely those of the authors and do not necessarily represent those of their affiliated organizations, or those of the publisher, the editors and the reviewers. Any product that may be evaluated in this article, or claim that may be made by its manufacturer, is not guaranteed or endorsed by the publisher.

## Supplementary Material

The updated supplementary material has been deposited to FigShare: doi: 10.6084/m9.figshare.15128094
